# Derived loss of signal complexity and plasticity in a genus of weakly electric fish

**DOI:** 10.1242/jeb.242400

**Published:** 2021-06-24

**Authors:** David E. Saenz, Tingting Gu, Yue Ban, Kirk O. Winemiller, Michael R. Markham

**Affiliations:** 1 Department of Ecology and Conservation Biology, Texas A&M University, College Station, TX 77843, USA; 2 Sam Noble Microscopy Laboratory, University of Oklahoma, Norman, OK 73019, USA; 3 Neurobiology Section, Biological Sciences Division, University of California, San Diego, La Jolla, CA 92093, USA; 4 Department of Biology, University of Oklahoma, Norman, OK 73019, USA; 5 Cellular & Behavioral Neurobiology Graduate Program, University of Oklahoma, Norman, OK 73019, USA

**Keywords:** Gymnotiform, Sodium channel, Melanocortin hormones, Adrenocorticotropic hormone

## Abstract

Signal plasticity can maximize the usefulness of costly animal signals such as the electric organ discharges (EODs) of weakly electric fishes. Some species of the order Gymnotiformes rapidly alter their EOD amplitude and duration in response to circadian cues and social stimuli. How this plasticity is maintained across related species with different degrees of signal complexity is poorly understood. In one genus of weakly electric gymnotiform fish (*Brachyhypopomus*), only one species, *B. bennetti*, produces a monophasic signal while all other species emit complex biphasic or multiphasic EOD waveforms produced by two overlapping but asynchronous action potentials in each electric organ cell (electrocyte). One consequence of this signal complexity is the suppression of low-frequency signal content that is detectable by electroreceptive predators. In complex EODs, reduction of the EOD amplitude and duration during daytime inactivity can decrease both predation risk and the metabolic cost of EOD generation. We compared EOD plasticity and its underlying physiology in *Brachyhypopomus* focusing on *B. bennetti*. We found that *B. bennetti* exhibits minimal EOD plasticity, but that its electrocytes retained vestigial mechanisms of biphasic signaling and vestigial mechanisms for modulating the EOD amplitude. These results suggest that this species represents a transitional phenotypic state within a clade where signal complexity and plasticity were initially gained and then lost. Signal mimicry, mate recognition and sexual selection are potential factors maintaining the monophasic EOD phenotype in the face of detection by electroreceptive predators.

## Abstract (precis)

**Highlighted Article:** In one electric fish genus, most species produce complex, plastic electric signals. One species that produces a simple signal shows reduced signal plasticity and retains vestigial mechanisms of signal complexity.

## INTRODUCTION

**Fig. 1.**
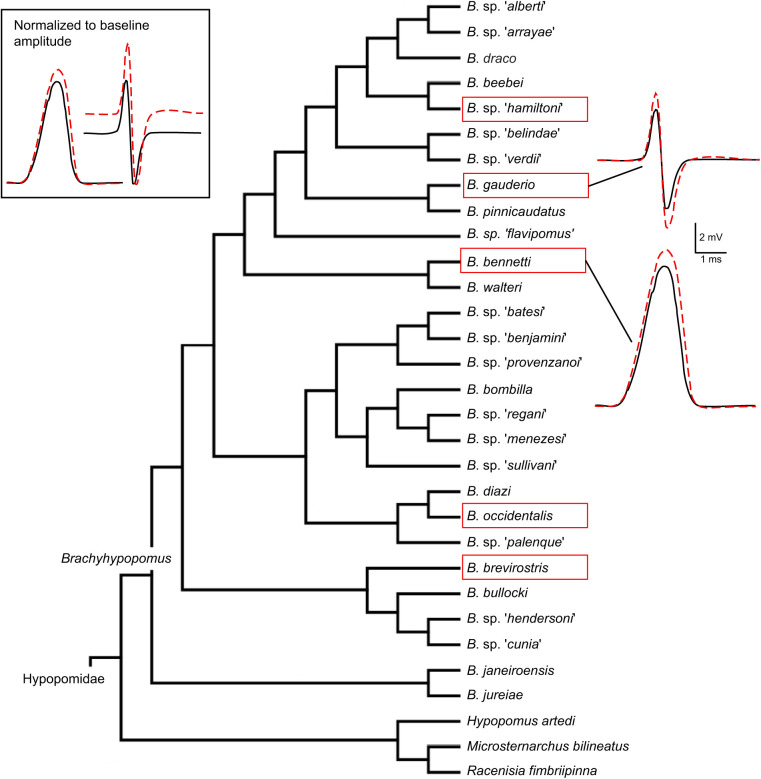
**Current phylogeny of *Brachyhypopomus*.** Species in red boxes are known to display circadian changes in electric organ discharge (EOD) waveform. *Brachyhypopomus occidentalis* was recorded by Hagedorn (1995). Insets on the right show the EOD waveforms of the biphasic EOD species *B. gauderio* and the monophasic EOD species *B. bennetti*; red dashed lines show nightly waveform changes. Inset on the left shows the same EOD waveforms, with amplitudes scaled to the same baseline amplitude. The baseline EOD amplitude is larger in *B. bennetti*; however, the magnitude of the EOD amplitude change is much smaller. Adapted with permission from Crampton et al. (2016b).

Gymnotiform fishes emit and detect weak electric fields to communicate with conspecifics and navigate in dark waters. The electric organ discharge (EOD) is produced by the near-simultaneous action potentials (APs) of excitable cells (electrocytes). Across their phylogeny, gymnotiforms have evolved diverse signals that vary in parameters such as discharge rate and waveform structure (Albert and Crampton, 2005). Despite a high species diversity and wide geographical range, species in the genus *Brachyhypopomus* (Hypopomidae) (Fig. 1) have been relatively well studied in terms of their evolutionary relationships (Crampton et al., 2016a, 2016b) and ecology (Waddell et al., 2019). The EODs have been examined in 18 of the 29 *Brachyhypopomus* species described. Twelve species have biphasic EODs, five have multiphasic EODs, and one species, *Brachyhypopomus bennetti*, has a derived monophasic EOD (Fig. 1; Sullivan et al., 2013; Crampton et al., 2016b).

Monophasy is a rare characteristic among weakly electric fishes, presumably because of its potential for detection by electroreceptive predators (Stoddard, 1999; Stoddard and Markham, 2008). The biphasic EOD is likely an adaptation to predation pressures from ‘eavesdropping’ electroreceptive predators (e.g. catfishes, electric eels, freshwater rays; Hanika and Kramer, 1999, 2000; Stoddard, 1999). The two EOD phases sum at a short distance away from the fish, attenuating the low-frequency DC component of the EOD detectable by the ampullary receptors of predators. Consequently, at greater distances, DC-balanced EODs are theoretically not detectable by electroreceptive predators (Stoddard, 1999; Stoddard and Markham, 2008; Stoddard et al., 2019). *Brachyhypopomus bennetti*'s monophasic EOD represents the asymmetrical extreme, with a large DC component and an amplitude 3–8 times larger than those of sympatric congeners (Crampton and Albert, 2006) that likely augment its detectability by electroreceptive predators. Stoddard (1999) suggested that *B. bennetti*'s EOD may function as a Batesian mimic of the EOD produced by the electric eel (*Electrophorus electricus*), the only gymnotiform capable of producing a discharge powerful enough to incapacitate prey and potential predators. Others have argued the likelihood of this mimicry and proposed additional hypotheses for the persistence of the monophasic phenotype (Crampton and Albert, 2006; Sullivan et al., 2013).

The impressive diversity of EOD waveforms found across the gymnotiform phylogeny is due in part to differences in electrocyte morphology as well as the diversity, kinetics and spatial distribution of the ion channels in these excitable cells (Markham, 2013). In some gymnotiforms, electrocyte ion channels can be regulated in such a way as to modify key EOD traits such as amplitude, waveform structure and low-frequency spectral content (Markham and Stoddard, 2005; Markham et al., 2009). These EOD properties can vary by sex and ontogeny and are likely important social cues (Markham and Stoddard, 2013). We refer to this modulation of EOD traits herein as EOD plasticity.

The genus *Brachyhypopomus*, is thought to be a specialist in this type of EOD plasticity. After the early discovery that *B. occidentalis* diurnally regulates its EOD amplitude (EODa) (Hagedorn, 1995), subsequent studies determined that *B. gauderio* also shows this behavior (Franchina and Stoddard, 1998), that both species exhibit EOD plasticity in response to social cues (Franchina et al., 2001), and that males especially may enhance the low-frequency content of the signal (Hagedorn and Zelick, 1989; Markham and Stoddard, 2005). Increasing EODa during nocturnal activity increases the metabolic cost as well as the active space of the signal, which carries implications for prey detection and for signal detection by both conspecifics and electroreceptive predators (Salazar and Stoddard, 2008; Stoddard and Markham, 2008; Stoddard et al., 2019). In *B. gauderio*, EODa is thought to be important for dominance displays and mate choice (Curtis and Stoddard, 2003; Gavassa et al., 2012b). Previous studies have determined that this EOD plasticity is regulated by melanocortin hormones, especially adrenocorticotropic hormone (ACTH) (Markham and Stoddard, 2005).

To better understand the function and physiology of EODa plasticity, we compared the effects of ACTH in three additional species of *Brachyhypopomus*, and focused on one species in particular, *B. bennetti*, the only congener that emits a monophasic EOD. We expected that *B. bennetti* would show EODa plasticity on the same scale as other *Brachyhypopomus* species because of its relatively large EODa (Crampton and Albert, 2006) and the potential conspicuousness of its monophasic EOD to electroreceptive predators (Stoddard, 1999; Stoddard and Markham, 2008). Additionally, we hypothesized that reversion to a monophasic EOD occurred in *B. bennetti* by the loss of voltage-gated Na^+^ channels and electrical excitability on the anterior face of its electrocytes, matching the general organization of electrocytes in another gymnotiform with a monophasic EOD, the electric eel (Ellisman and Levinson, 1982; Fritz et al., 1983). We were surprised to find that *B. bennetti* exhibited only minor EODa plasticity and, more unexpectedly, that the anterior membrane of *B. bennetti* electrocytes expresses voltage-gated Na^+^ channels and is electrically active.

## MATERIALS AND METHODS

### Animals

A limitation to working with this system is that it can be difficult to reliably and responsibly acquire specific species. At this time, *B. gauderio* is the only species of *Brachyhypopomus* dependably bred in captivity. Individuals of *B. bennetti* (Sullivan et al., 2013), *B*. cf. *hamiltoni* (Crampton et al., 2016b) and *B. brevirostris* (Steindachner 1868) were wild caught from Manaus, Brazil, and exported in collaboration with researchers at the Brazilian Institute of Amazonian Research under ICMBio authorizations #14833 and #14834. These fish can be highly sensitive to the stressors of transport so ultimately we had a total of 8 individuals of *B. bennetti* and 1 *B. brevirostris* for *in vitro* experiments. Specimens of *B. gauderio* Giora & Malabarba 2009 were captive bred from colonies maintained at the University of Oklahoma. All methods were approved in advance by the Institutional Animal Care and Use Committees of Texas A&M University and the University of Oklahoma.

### Immunohistochemistry

We immunolabeled voltage-gated Na^+^ channels and axon terminals in the electric organ using antibodies and protocols described previously (Ban et al., 2015). We labeled voltage-gated Na^+^ channels with an Anti-Pan Na_v_ antibody (1:100, Lot 23, Alomone Labs, Jerusalem, Israel) that has been validated in electric fish (Ban et al., 2015), and we labeled axon terminals with 3A10 (1:100, developed by T. Jessel and J. Dodd, and obtained from DSHB, Antibody Registry ID: AB_531874). For *B. bennetti* tissue (*n*=2 fish), selected sections were imaged on a Leica SP8 confocal laser scanning microscope using a 20×0.75 NA oil objective. A GaN 405 laser with a 405 nm laser line was used to excite DAPI with an emission detection window between 415 and 455 nm. Similarly, an argon laser line was used to excite the Alexa Fluor 488 and an emission detection window was set at 550–5500 nm. *Z*-series of images were acquired via sequential scanning. For *B. gauderio* tissues, immunolabeled sections were imaged on a Zeiss ApoTome.2 microscope with 5×0.16 NA, 10×0.45 NA and 20×0.80 NA dry objectives. Images were acquired using a Zeiss AxioCam MRm and then processed by Zeiss AxioVision Rel.4.8. We created optical sections of the fluorescent samples using structured illumination. Image contrast was adjusted in Fiji and Adobe Photoshop for better visualization of fine structures in the electrocytes.

### Injections and recordings

EODs were recorded using a pair of silver electrodes on either side of the fish. EODs were amplified using a BMA 200 AC/DC Bioamplifier (CWE, Inc.) and digitized at 16 bit resolution using an A/D converter (CE Data translation USB Data Acquisition) at a sampling rate of at least 50 kHz. Baseline EOD recordings were made for a minimum of 30 min. Fish were then quickly removed from the tank and given an intramuscular injection (1 µl g^−1^) of either 30 µmol l^−1^ ACTH or normal saline in a process that took less than 15 s.

### Circadian recordings

Circadian recordings were conducted using an automated system for recording calibrated EODs round the clock from freely swimming fish described previously by Stoddard et al. (2003). EODs were recorded at ∼60 s intervals for at least 3 days to assess circadian variation in EOD waveform.

### Solutions for *in vivo* injections and *in vitro* electrophysiology

The normal saline contained 114 NaCl, 2 KCl, 4 CaCl_2_·2H_2_O, 2 MgCl_2_·6H_2_O, 5 Hepes, 6 glucose; pH to 7.2 with NaOH. For some *in vitro* experiments with *B. bennetti* electrocytes, NaCl was replaced with choline chloride to reduce the Na^+^ concentration to 14.5 mmol l^−1^, and pancuronium bromide (1 µmol l^−1^) was added to the saline. ACTH (from porcine pituitary) was purchased from Sigma Aldrich (St Louis, MO, USA). Tetraethyl ammonium (TEA), barium chloride and pancuronium bromide were obtained from Millipore-Sigma. Collagenase (type IV, Worthington Biochemical, Lakewood, NJ, USA) was prepared in normal saline.

### Electrophysiology

**Fig. 2.**
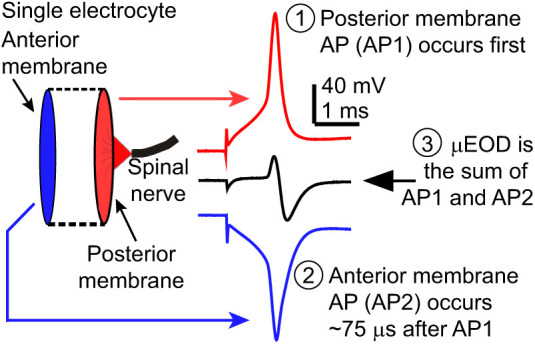
**Schematic diagram of the physiology of biphasic single-cell discharge (µEOD) production in *Brachyhypopomus* electrocytes.** Electrocytes are large disc-shaped cells with regions of excitable membrane on the posterior and anterior membranes. The biphasic μEOD is produced by two action potentials (APs) generated in close succession on these membranes. When the spinal nerve activates the electrocyte's cholinergic synapse, the posterior membrane fires first (AP1, red) followed approximately 75 μs later by an AP on the non-innervated anterior membrane (AP2, blue). AP2 is inverted in this figure to reflect the fact that the ionic currents producing AP2 are directed in the opposite direction to those that produce AP1. These two APs sum to produce the biphasic μEOD (black).

*Brachyhypopomus* electrocytes are large disc-shaped cells innervated on the posterior side (Bennett, 1961, 1971; Trujillo-Cenóz et al., 1984; Markham, 2013). The biphasic EOD is the result of two APs (Fig. 2). The first AP (AP1) occurs at the posterior face of the electrocytes in the direction of the head, and the second (AP2) occurs at the anterior face in the direction of the tail. The summation of the APs in a single electrocyte produces a biphasic single-cell discharge (µEOD), and the whole EOD is the sum of the composite µEODs.

For electrophysiological recordings from single electrocytes, we removed a small piece of the tail (∼1 cm) and carefully dissected the skin off to expose the electric organ. The tissue was pinned to a Sylgard-coated recording dish and incubated in saline with 2% collagenase for 45 min to weaken the tissue surrounding the electrocytes. The preparation was then flushed several times with normal saline at room temperature (23±1°C) over a period of at least 15 min before recording. Intracellular stimulation and recordings used an Axoclamp 900 A amplifier (Molecular Devices, Union City, CA, USA) and extracellular recordings were completed using a Dagan TEV200A amplifier (Dagan Corp, Minneapolis, MN, USA) in current clamp mode. A Digidata 1440 interface and PClamp 10.0 software (Molecular Devices, San Jose, CA, USA) were used to control all amplifiers. Data were sampled and digitized at 100 kHz sampling rate. Extracellular pipettes were pulled from 1.2 mm thin-wall borosilicate glass and had a resistance between 400 and 600 kΩ when filled with normal saline. Intracellular pipettes had a resistance of 0.8–1.2 MΩ when filled with 3 mol l^−1^ KCl.

#### Current clamp

We used a multi-electrode current clamp procedure to record APs and µEODs from single electrocytes as described in more detail elsewhere (Bennett, 1961; Markham and Stoddard, 2005; Markham and Zakon, 2014). Briefly, one intracellular pipette delivered a depolarizing current step to elicit the µEOD. A second intracellular pipette recorded the intracellular potential and two extracellular pipettes placed within 50 µm of the posterior and anterior membranes recorded extracellular potentials, one from each membrane. Off-line subtraction of the posterior extracellular record and the anterior extracellular record from the intracellular record result in AP1 and AP2, respectively. Subtraction of the posterior extracellular record from the anterior extracellular record returns the µEOD.

Only electrocytes with stable resting potentials <80 mV and input resistances were recorded. Once all the electrodes were in place, we delivered 6 ms depolarizing current steps while manually adjusting the stimulus current magnitude until a µEOD was dependably elicited. A baseline recording was made of µEODs every 60 s for at least 15 min, after which we perfused normal saline for control cells or normal saline containing 100 nmol l^−1^ ACTH. Because of the limited number of specimens, the timing of ACTH application and the length of the baseline recordings were staggered between individuals as an added measure of control for non-specific effects. Solutions were changed during the interstimulus interval with a quick perfusion of 5 ml followed by slow continuous perfusion at 5 ml h^−1^. We recorded µEODs at 60 s intervals for the remainder of the experiment.

#### Two-electrode voltage clamp

Holding potential for all cells was −90 mV. Whole-cell currents were recorded in normal saline in response to voltage steps from 140 mV to 0 mV in 5 mV increments. Na^+^ currents (*I*_Na_) were recorded in reduced-Na^+^ saline (14.5 mmol l^−1^ NaCl) with 2 mmol l^−1^ BaCl_2_ and 10 mmol l^−1^ TEA to block any K^+^ conductance, and 1 µmol l^−1^ pancuronium bromide to prevent spontaneous contraction of tail muscles. Voltage clamp protocols to assess *I*_Na_ activation and inactivation consisted of a 50 ms conditioning step to −120 mV followed by 20 ms voltage steps from −120 to 25 mV in 5 mV increments and then a 20 ms step to 0 mV. Recovery of *I*_Na_ from inactivation was assessed with a protocol that consisted of a 50 ms conditioning step to −120 mV followed by an activation step to 0 mV and a recovery step to −120 mV for 0.5–12.5 ms in 0.5 ms increments and then a step to a test potential of 0 mV for 20 ms.

### Data treatment and analysis

For *in vivo* experiments, paired *t*-tests were used to compare percentage change in EOD parameters (relative to baseline) between ACTH and saline. The same was done for the current clamp data. Statistical analyses were not performed for the injection data on *B.* cf. *hamiltoni* or for current clamp data on *B. brevirostris* because only one individual was available for each experiment (see Results for modified procedure). Electrophysiology data were analyzed with Clampfit 10.7.0 (Molecular Devices) and Matlab (Mathworks, Natick, MA, USA).

### Computational simulations

For numerical simulations, we modeled the electrocyte as three cylindrical compartments arranged as a passive central compartment coupled to two flanking active compartments. Anterior membrane capacitance and Na^+^ conductance density were the only parameters manipulated in the model. All other parameters were constrained in accordance with experimental data. External stimulation current was applied only to the central compartment, consistent with experimental procedure. The capacitance *C*_C_ for the central compartment was 100 nF and the capacitance of the posterior and anterior compartments, *C*_P_ and *C*_A_ respectively, was 50 nF, yielding a total membrane capacitance of 150 nF, consistent with empirical measurements of whole-cell capacitance. Differential equations were coded and integrated with Matlab using Euler's method with integration time steps of 1×10^−9^ s. All model parameters are shown in Table 1.

**Table 1.**
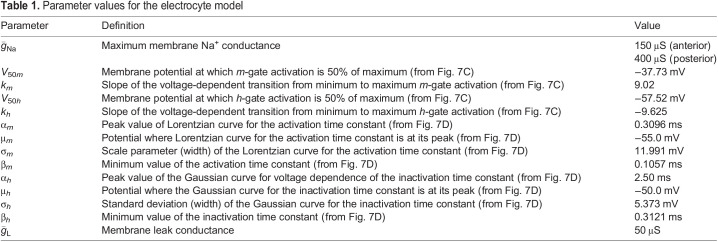
Parameter values for the electrocyte model

The passive central compartment's current balance equation included only terms representing the injected stimulation current pulses [*I*_Stim_(*t*)], passive leak (*I*_LC_) and coupling to the two active compartments:(1)
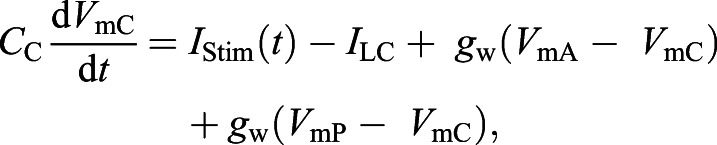
where *g*_w_ is the coupling conductance, fixed at 3.33 μS for all compartments, *V*_m_ is the membrane potential (with subscripts C, A and P indicating the central, anterior and posterior compartments) and *I*_LC_ is the passive leak current in the central compartment given by the equation:(2)
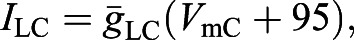
where 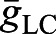
=5 µS.

The current balance equations for the anterior and posterior active compartments were, respectively:(3)
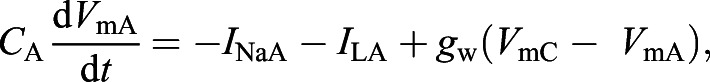
(4)
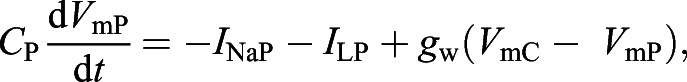
where *V* is the membrane potential, *I*_Na_ represents Na^+^ current and *I*_L_ is the leak current. Equations for these currents in the anterior and posterior compartments were as follows:(5)
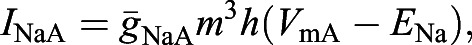
(6)
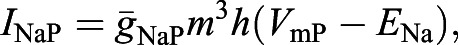
(7)
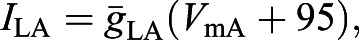
(8)
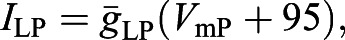
where *m* is the activation variable for the activation (m-)gates, *h* is the activation variable of the inactivation (h-)gates and *E*_Na_ is the Na^+^ equilibrium potential.

The gating variables in Eqns 5 and 6 evolve by Eqn 9 where *j*=*m* or *h*:(9)
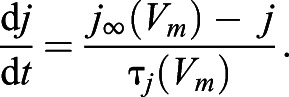
The values of *j*_∞_ were determined in a voltage-dependent fashion as follows:(10)
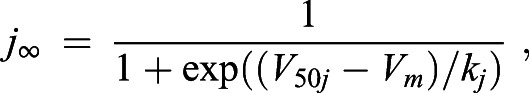
where *V*_50_*_j_* and *k_j_* are derived from Boltzmann sigmoidal fits to experimental data and are given in Table 1. The voltage-dependent time constant of activation or inactivation τ*_j_* is given by Eqn 11 for *j*=*m*:(11)
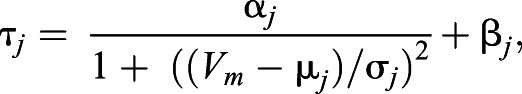
and by Eqn 12 for *j*=*h*:(12)
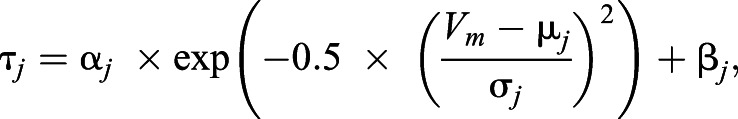
where values of α*_j_*, β*_j_*, µ*_j_* and σ*_j_* were determined by least-squares best fits to our experimental voltage-clamp data and are given in Table 1.

## RESULTS

### Immunolocalization

**Fig. 3.**
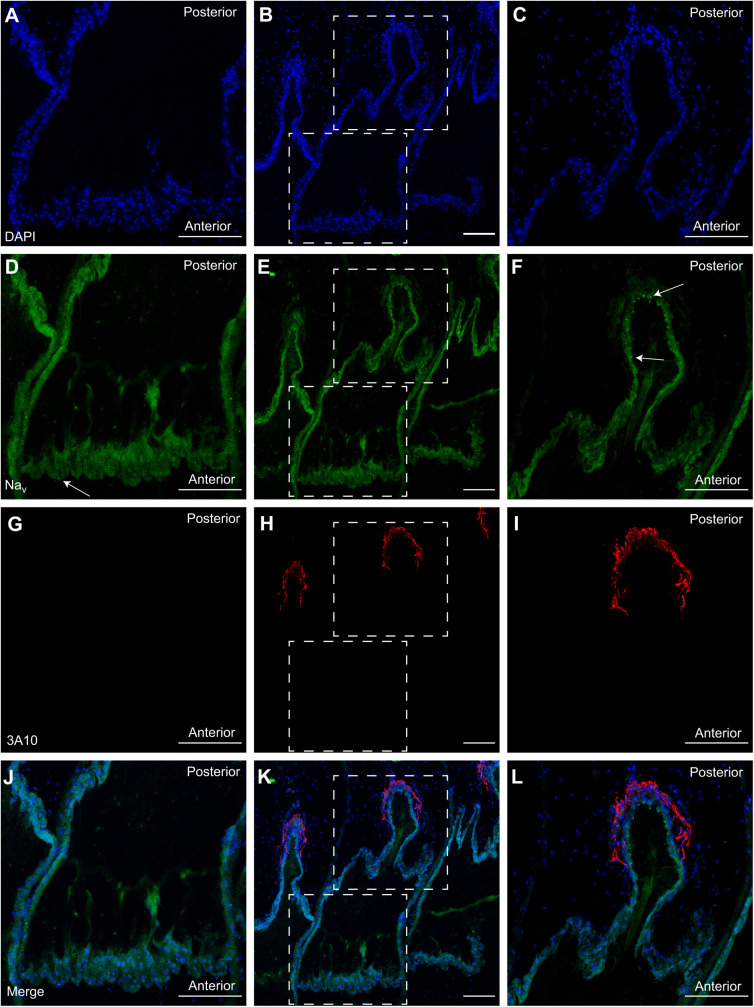
**Immunolabeling of the axon terminal and voltage-gated Na^+^ channels in electrocytes of the monophasic EOD species *B. bennetti*.** The center column shows the full cell. Dashed boxes delineate enhanced images of the anterior membrane (bottom box) shown in the left column and the posterior membrane (top box) shown in the right column. (A–C) DAPI labels the electrocyte nuclei and provides a relative outline of electrocytes. (D–F) Anti-Pan Na_V_ labels Na^+^ channels. Punctate fluorescence signal is visible on both membranes as well as on internal structures. (G–I) 3A10 labels the axon terminal on the stalk. (J–L) Merged images of the three previous rows. Scale bars: 100 µm.

**Fig. 4.**
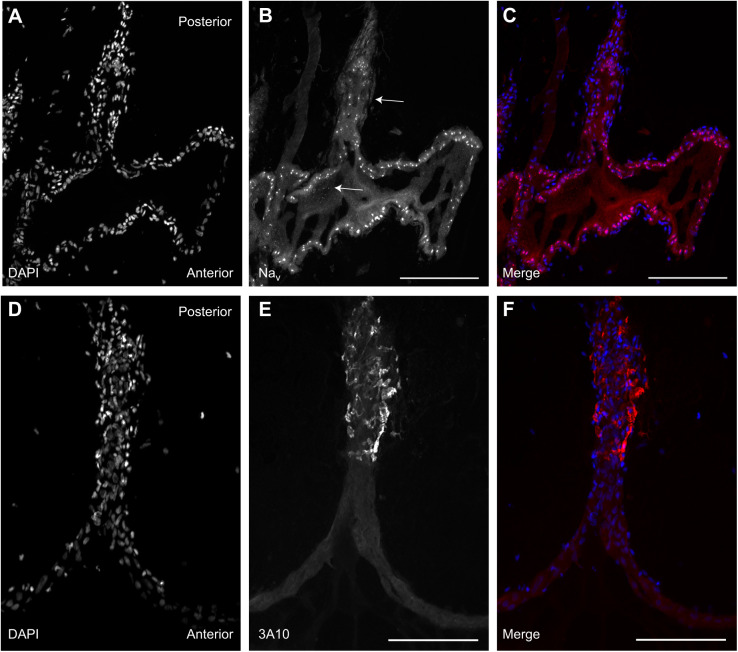
**Immunolabeling of the axon terminal and voltage-gated Na^+^ channels in electrocytes of the biphasic EOD species *B. gauderio*.** (A) DAPI labeling of nuclei provides the general outline of the electrocyte. (B) Anti-Pan Na_V_ labels Na^+^ channels. Arrows highlight puncta present on internal structures. (C) Merge of A and B. (D) DAPI labels the nuclei outlining the stalk of a different cell. (E) 3A10 labels the axon terminal on the stalk. (F) Merge of D and E. Scale bars: 100 µm.

**Fig. 5.**
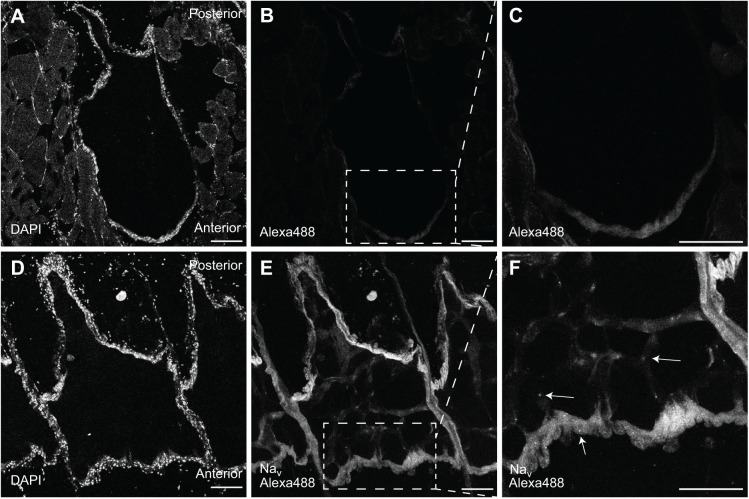
**Na^+^ channels are present in both anterior and posterior membranes of the electrocytes in the monophasic EOD species *B. bennetti*.** (A,D) DAPI labels the electrocyte nuclei and provides a relative outline of electrocytes with the caudal stalk prominently visible. (B,C) Control cells labeled with only the secondary antibody (Alexa Fluor 488) show minimal autofluorescence. (E,F) Binding of the primary antibody Anti-Pan Na­_V_ indicates the presence of voltage-gated Na^+^ channels in the electrocyte membrane. The high magnification image in F reveals a punctate signal (arrows) on the anterior membrane, contrary to our expectations; this is also visible on internal structures of electrocytes. Scale bars: 100 µm.

Immunohistochemistry confirmed that electrocytes are innervated on the posterior stalk by spinal electromotor neurons in *B. bennetti* and *B. gauderio* (Fig. 3G–L and Fig. 4E,F, respectively). As expected, in *B. gauderio*, Na^+^ channels were found on both the posterior and anterior membranes (Fig. 4A–C). Contrary to our expectations, Na^+^ channels were also found on both the posterior and anterior membranes of *B. bennetti* (Fig. 3D–F and Fig. 5). Interestingly, Na^+^ channels were also present on internal cell structures (Figs 4B, 5F). Because of the limited availability of specimens, we were unable to quantify Na^+^ channel expression because *B. bennetti* electrocytes are large (∼600–900 µm in length) and the labeling of Na^+^ channels varies depending on where the cells were sectioned.

### Effects of ACTH *in vivo*

We found that intramuscular injection of ACTH significantly increased the peak-to-peak EODa in the biphasic species *B. brevirostris*, and in *B.* cf*. hamiltoni* (33.7±4.7% and 16.1%; *n*=6 and 1, respectively; data for *B*. cf. *hamiltoni* not shown), as has been shown for *B. gauderio* (Markham and Stoddard, 2013). Separate evaluation of the phases in *B. brevirostris* showed that the amplitude of both P1 and P2 increased (*t*=2.23, *P*=0.056). On average, ACTH also increased EODa in *B. bennetti*; however, the effect was considerably weaker (*t*=2.45, *P*=0.094; *n*=6). Saline controls slightly decreased the EODa in all four species. The duration of both P1 and P2 (measured at 2% of the peak amplitude) increased in *B. brevirostris* (*P*<0.001, *t*=2.57) and *B.* cf*. hamiltoni* (data not shown). In *B. bennetti*, the duration of the EOD did not change appreciably.

### Effects of ACTH *in vitro*

**Fig. 6.**
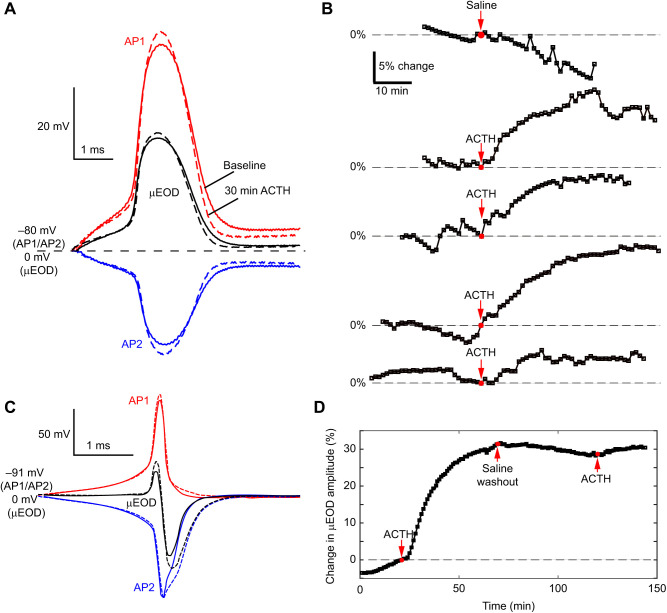
**Electrocyte APs and response to ACTH in *B. bennetti* and *B. brevirostris* electrocytes*.*** (A) Representative recordings from a *B. bennetti* electrocyte of AP1 (red) and AP2 (blue) with the resulting µEOD (black). Dashed lines show the APs and µEOD 30 min after perfusion of ACTH (*n*=6). (B) Recordings showing percentage change in µEOD amplitude for five different *B. bennetti* individuals, after perfusion of either a saline control (*n*=1 individual) or ACTH (*n*=4 individuals). (C) Recordings from a *B. brevirostris* electrocyte of AP1 (red) and AP2 (blue) with the resulting µEOD (black). Dashed lines show the action potentials and µEOD 30 min after perfusion of ACTH (*n*=13). (D) Percentage change in µEOD amplitude for one *B. brevirostris* electrocyte in response to ACTH (*n*=12).

Using multi-electrode current clamp, we measured µEOD parameters from individual electrocytes of *B. brevirostris* and *B. bennetti*. As previous studies have shown in *B. gauderio* (Markham and Stoddard, 2005; Markham et al., 2009, 2013), ACTH increased the amplitude of both P1 and P2 of the µEOD in *B. brevirostris* and, concurrently, the total µEOD amplitude (Fig. 6C,D), the duration of P2 and the AP1–AP2 delay (data not shown). Because only one individual was available for testing, we attempted to wash out the ACTH after 30 min with saline (60 min of the total recording), during which we observed a slow decrease in µEOD amplitude over the course of the next hour. We then reapplied ACTH and saw a small increase again in the µEOD amplitude (Fig. 6D). In *B. bennetti*, ACTH also increased the amplitude of the µEOD (Fig. 6A,B; *n*=4); however the effect was much smaller relative to *B. gauderio* and *B. brevirostris*. Again, contrary to our expectations, we observed APs on both the posterior and the anterior membrane (Fig. 6A). Perfusion of ACTH slightly increased the amplitude of AP1, AP2 and the µEOD. No other parameters showed consistent changes in response to ACTH.

### Ionic currents in *B. bennetti* electrocytes

**Fig. 7.**
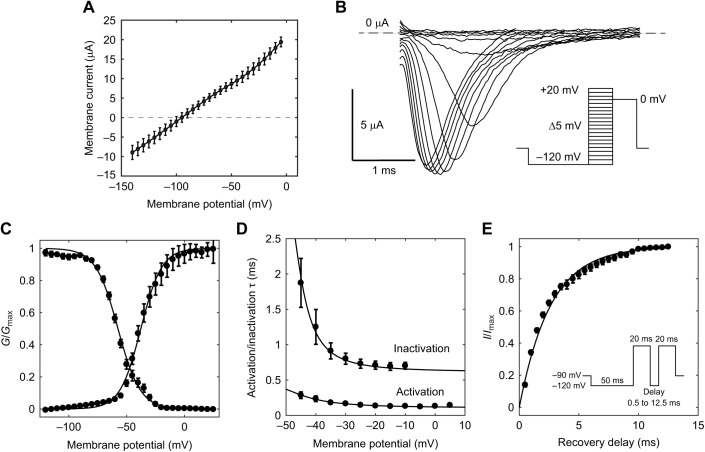
**Characterization of *B. bennetti* Na^+^ currents.** (A) Whole-cell *I*–*V* relationships at steady state in *B. bennetti* electrocytes are linear and show no evidence of significant inward- or outward-rectifying K^+^ currents. (B) Representative currents elicited by voltage steps from −115 to 20 mV in 5 mV increments. Inset shows the voltage command protocol, which was also used for data shown in C and D. (C) Activation and inactivation *G*–*V* plots for Na^+^ currents. Solid lines are Boltzmann sigmoidal fits. (D) Activation and inactivation τ–*V* curves (where τ is the time constant of recovery from activation/inactivation). Solid line is a Gaussian fit for inactivation data and a Lorentzian fit for activation data. (E) *I*_Na_ recovery from inactivation. Solid line represents a single-exponential fit. Inset shows the voltage command protocol. Data in A and C–E are means±s.e.m.

Whole-cell currents recorded in voltage clamp showed an extremely large transient inward current that could not be controlled by the amplifier even at its maximum gain (50,000 *V*/*V*). Currents at steady state could be controlled by the amplifier's steady-state gain (10^6^ *V*/*V*). Steady-state *I*–*V* relationships were linear, showing no evidence of meaningful inward- or outward-rectifying K^+^ conductance (Fig. 7A).

Given the apparent absence of significant voltage-gated K^+^ currents and the paucity of subject animals, we focused on describing Na^+^ conductance in *B. bennetti* electrocytes because this is apparently the dominant ionic conductance shaping the electrocyte APs. We used a reduced-Na^+^ extracellular saline solution (14.5 mmol l^−1^ NaCl) to reduce the driving force on *I*_Na_ and allow stable voltage-clamp control during *I*_Na_ activation. *Brachyhypopomus bennetti* electrocytes showed transient inward Na^+^ currents (Fig. 7B,C) with typical voltage dependence of activation and inactivation time constants (Fig. 7D). The time constant of recovery from inactivation (τ=3.80 ms; Fig. 7E) is the slowest for any gymnotiform species thus far reported, by a factor of four (cf. Ferrari et al., 1995; Markham et al., 2013; Markham and Zakon, 2014). This is not completely unexpected considering *B. bennetti* has a very low EOD frequency (<10 Hz).

### Computational electrocyte simulations

**Fig. 8.**
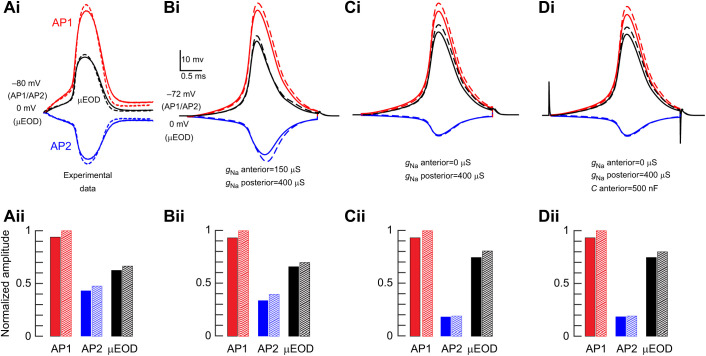
**Computational simulations of *B. bennetti* electrocytes compared with experimental data.** Red lines and bars represent the posterior compartment (AP1), blue lines and bars represent the anterior compartment (AP2) and black lines represent whole-cell µEODs. (Ai) Experimental recordings of *B. bennetti* AP1, AP2 and µEOD reproduced from Fig. 6A. Solid lines are baseline recordings and dashed lines represent APs and µEODs 30 min after application of ACTH. (Aii) Amplitude of APs and µEODs in Ai normalized to the amplitude of AP1 after ACTH treatment. Solid bars correspond to solid lines in Ai and hatched bars correspond to dashed lines in Ai. (Bi–Di) Simulations of electrocyte APs and µEODs based on experimental measurements of Na^+^ conductance parameters under voltage clamp. Stimulation is a 3 ms step current of 4400 nA (not shown). Passive membrane responses to stimulus current are not shown. Solid lines represent baseline simulations and dashed lines show simulations after Na^+^ conductance was increased by 10% in both the anterior and posterior compartments. (Bii–Dii) Amplitude of APs and µEODs in Bi–Di normalized to the amplitude of AP1 after the increase in Na^+^ conductance in each case. Solid bars in Bii–Dii correspond to solid lines in Bi–Di and hatched bars in Bii–Dii correspond to dashed lines in Bi–Di. (B) Responses of the baseline model electrocyte with all parameters as in Table 1. Note that AP and µEOD amplitudes closely resemble experimental recordings in A. (C) Responses of a model electrocyte where the anterior Na^+^ conductance (*g*_Na_) is eliminated. (D) Responses of a model electrocyte where the anterior Na^+^ conductance is eliminated and the anterior compartment capacitance (*C* anterior) is increased by a factor of 10 to a value of 500 nF.

With parameters derived from our voltage-clamp data, we simulated *B. bennetti* electrocytes to evaluate whether our experimentally observed Na^+^ channel localization, Na^+^ conductance and electrocyte passive properties are sufficient and necessary to reproduce the electrocyte APs recorded *in vitro* and to reproduce the effects of ACTH observed during our current-clamp recordings. A model electrocyte that included Na^+^ conductance in both the posterior and anterior compartments produced simulated AP1, AP2 and µEOD waveforms that were strikingly consistent with experimentally recorded waveforms (Fig. 8A,B). Increasing the Na^+^ conductance by 10% in both the anterior and posterior compartments increased AP1, AP2 and µEOD amplitude in the model cell in a manner nearly identical to the effects of ACTH recorded *in vitro* (Fig. 8A,B).

To test whether Na^+^ conductance in the anterior compartment is necessary to reproduce our experimental results, we evaluated the performance of two alternative model electrocytes. In one model, we eliminated the anterior compartment Na^+^ conductance. This model failed to reproduce experimentally recorded AP1, AP2 and µEOD waveforms, as well as the observed effects of ACTH (Fig. 8C), even across a broad range of posterior compartment Na^+^ conductance densities (data not shown). In a second alternative model, we eliminated the anterior compartment Na^+^ conductance, but increased the anterior compartment capacitance up to tenfold in order to test an earlier proposal that AP2 is a result of capacitive discharge from the anterior membrane (Bennett, 1971). This model also failed to reproduce experimental findings (Fig. 8D) even across a broad range of anterior compartment capacitances and posterior compartment Na^+^ conductance densities (data not shown). Taken together with our experimental data, these simulations support the conclusion that both anterior and posterior compartments of *B. bennetti* electrocytes are electrically active and endowed with voltage-gated Na^+^ channels.

**Fig. 9.**
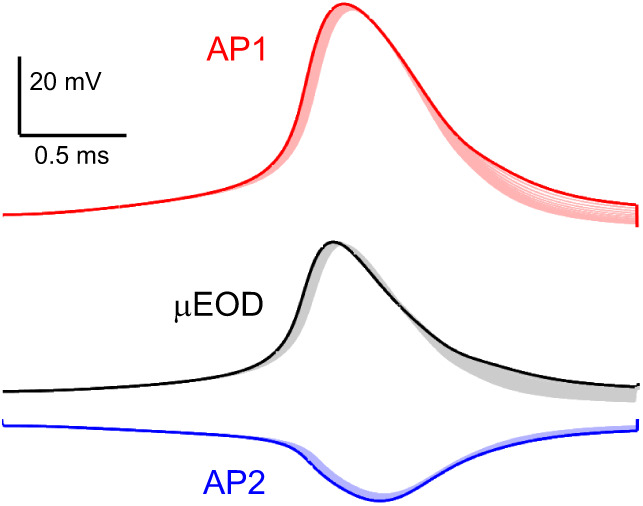
**Computational simulations of *B. bennetti* electrocytes with delayed-rectifier K^+^ conductance.** Red lines represent the posterior compartment, blue lines represent the anterior compartment and black lines represent whole-cell µEODs. The baseline simulation (bold lines) is the same simulation as in Fig. 8B. A simulated delayed-rectifier K^+^ conductance based on experimental data from the biphasic gymnotiform *Steatogenys elegans* (Markham and Zakon, 2014) was added to the anterior and posterior electrocyte compartments. Thin lines represent 100 simulations that include all combinations of anterior and posterior compartment K^+^ conductance levels ranging from 400 µS to 4000 µS in 400 µS increments, yielding a total of 100 parameter combinations. Note that minor changes in AP duration do not yield significant differences in the µEOD.

Voltage-gated K^+^ conductance plays an important role in shaping AP duration and timing in other biphasic gymnotiforms (Markham, 2013; Markham and Zakon, 2014). Here, we did not observe K^+^ conductance with voltage dependence and kinetics sufficient to shape AP waveform and timing in *B. bennetti* electrocytes (Fig. 7A). However, because our quantitative voltage-clamp analyses were restricted to Na^+^ conductance, we cannot conclusively rule out a role of K^+^ conductance based on experimental data. Instead, we tested the potential role of K^+^ conductance in *B. bennetti* electrocytes by adding to the anterior and posterior compartments a simulated delayed-rectifier K^+^ conductance based on experimental data from the biphasic gymnotiform *Steatogenys elegans* (Markham and Zakon, 2014). We tested combinations of anterior and posterior compartment K^+^ conductance levels ranging from 400 to 4000 µS in 400 µS increments, for a total of 100 parameter combinations. The model results showed only minor effects of such K^+^ conductance on the AP and µEOD durations, with little to no effect on AP and µEOD amplitude (Fig. 9). These findings indicate that *B. bennetti* electrocytes indeed may not depend on voltage-gated K^+^ conductance to regulate AP and µEOD outputs.

## DISCUSSION

Although our sample size was limited, multiple approaches and sources of evidence (*in vivo* recordings, *in vitro* electrophysiology, immunolabeling and computational simulations) all converged on the same striking observations: *B. bennetti* shows greatly reduced EOD plasticity compared with other *Brachyhypopomus* species and both the anterior and posterior membranes of *B. bennetti* electrocytes are electrically active. These findings challenge previous assumptions as to the role of signal complexity in hypopomid life histories and engender new questions about the evolution and energetics of hypopomid electrocytes.

### EOD plasticity in *Brachyhypopomus*

Previous studies have shown that *B. gauderio* significantly modifies its EOD waveform in response to circadian and social cues via direct action of melanocortin peptide hormones on the electrocytes (Markham and Stoddard, 2005, 2013). Circadian plasticity of the EOD waveform has also been reported in *B. occidentalis* (Hagedorn, 1995). Here, we report similar ACTH-mediated EOD plasticity in three additional species of *Brachyhypopomus* (*B. bennetti*, *B. brevirostris* and *B.* cf. *hamiltoni*). Relative to the other congeners studied, *B. bennetti* exhibited minimal EOD plasticity.

The extent of this EOD plasticity and its regulation vary by sex and ontogeny in *B. gauderio* (Markham and Stoddard, 2013). Though our sample size is insufficient to confirm demographic variation for the species tested here, it is interesting that the plasticity seems consistent throughout the genus (Fig. 1) with *B. bennetti* as an exception. While some fishes from other gymnotiform genera exhibit ACTH-mediated EODa plasticity (McAnelly and Zakon, 1996; Markham et al., 2009), others do not (D.E.S., unpublished data). The function of this plasticity is not yet fully understood, but two probable roles are social and energetic. The active electrosensory system can be metabolically costly. Estimates of EOD production costs range from 4% to 22% of the daily metabolic budget in species with relatively slow EOD repetition rates such as *B. gauderio* (Salazar and Stoddard, 2008) and may cost well over 30% in species with higher repetition rates such as *Eigenmannia virescens* (Sternopygidae) (Lewis et al., 2014). Previous studies have reasonably argued that circadian regulation of EODa is adaptive for conserving energy. Therefore, it is interesting that *B. bennetti* shows decreased EODa plasticity, especially considering that its EODa is 3–8 times larger than those of sympatric congeners (Crampton and Albert, 2006), and the present results suggest that the physiology of EOD production is energetically inefficient in this species.

Another surprising finding was the magnitude of AP2 in *B. bennetti* electrocytes and the presence of voltage-gated Na^+^ channels on the anterior membrane. The current-clamp data suggest that, while the EOD of *B. bennetti* is monophasic, the µEOD is still composed of two APs. Because these APs completely overlap, the discharge from the anterior face (AP2) negates a significant fraction of AP1, potentially wasting considerable energy. The apparent absence of K^+^ conductance in *B. bennetti* electrocytes may be, in part, responsible for the complete overlap of AP1 and AP2. Bennett (1971) recorded electrocytes from a monophasic hypopomid, likely *B. bennetti*, but argued that the anterior membrane is electrically inexcitable. Instead, it was suggested that a smaller spike from the uninnervated membrane is a discharge of the membrane's capacitance, similar to what is suggested to occur in the uninnervated face of electrocytes in *Gymnarchus niloticus*, an African weakly electric fish (Bennett, 1971; Schwartz et al., 1975)*.* Our finding that voltage-gated Na^+^ channels are indeed present on the anterior face, in conjunction with our electrophysiology data and computational simulations, suggests the anterior membrane is, in fact, excitable.

Larval *Brachyhypopomus* begin generating monophasic EODs and transition to more complex waveforms as they grow (Franchina, 1997; Crampton et al., 2016b). This is thought to be the case for all gymnotiform fishes, and a monophasic EOD is generally considered to be the ancestral condition (Stoddard, 2002); however, this has not been confirmed because of unresolved phylogenetic relationships of families (Arnegard et al., 2010; Lovejoy et al., 2010; Tagliacollo et al., 2016; Alda et al., 2018). The presence of Na^+^ channels and generation of AP2 on the anterior membrane provides evidence against the assumption that *B. bennetti*'s monophasic EOD is a retention of the pedomorphic monophasic condition. Is this electrical excitability of the anterior membrane a costly vestige of *B. bennetti*'s biphasic ancestry or does AP2 serve some unknown function? While the sample sizes of the *in vitro* assays are admittedly small, it is nonetheless compelling that multiple lines of evidence from each assay point to the same conclusion: *B. bennetti* electrocytes have Na^+^ channels on the anterior membrane that contribute to a second membrane depolarization of significant magnitude. Consequently, AP2 decreases the energetic efficiency of the EOD, without the benefit of producing a biphasic EOD to cloak the EOD from electroreceptive predators.

### The persistence of the monophasic EOD

As briefly reviewed in the Introduction, previous evidence suggests that monophasic EODs should be more detectable by electroreceptive predators (Hanika and Kramer, 1999, 2000; Stoddard, 1999; Stoddard and Markham, 2008; Stoddard et al., 2019). Given that the relatively large amplitude of *B. bennetti*'s monophasic EOD could further augment its detectability by electroreceptive predators, the persistence of this trait is interesting.

Previous studies have suggested that *B. bennetti*'s EOD functions as a Batesian mimic of the electric eel's monophasic EOD (Stoddard, 1999), as the eel is the only gymnotiform capable of producing a strong electric discharge to incapacitate predators and prey alike. Crampton and Albert (2006) argue that this mimicry is not a likely evolutionarily stable strategy because of a large discrepancy in the relative abundance of *B. bennetti* and *E. electricus*, the former being three orders of magnitude more abundant than the latter. Instead, Crampton and Albert (2006) suggest the persistence of the monophasic EOD is related to species recognition.

Sensory bias and a female preference for signals with low-frequency energy could further explain the reversion to a monophasic EOD. Sexual dimorphism has been documented in many gymnotiform species*.* According to Crampton and Albert (2006), *B. bennetti* males produce EODs with amplitudes 2–3 times larger than those of females of comparable size, likely because they possess relatively larger sized electrocytes compared to juveniles and females (Crampton et al., 2016b). Building on the hypothesis of compatible mate (‘species’) recognition, it is possible that the monophasic EOD of *B. bennetti* could be a result of sexual selection. Consistent with the handicap principle, female preference for a large amplitude could select for a costly signal in terms of both energetics and susceptibility to predation (Zahavi, 1975). A mate choice study on *B. gauderio* suggests that EODa is the most salient EOD feature used by females to assess potential mates (Curtis and Stoddard, 2003). Maximum EODa is largely a function of fish size, with larger individuals having more electrocytes thus producing larger EODs. Larger amplitudes also imply greater metabolic costs (larger APs require more ATP to restore electrocyte membrane potential per EOD), meaning a large amplitude could serve as an honest indicator of male condition. Still, mate choice studies with *B. bennetti* are needed to test this potential preference.

### Conclusion

A limitation of the experiments reported here is the restricted sample size, a sometimes unavoidable consequence of collecting specimens in the wild. A further difficulty when working with specific species of gymnotiforms is that they are rarely, if ever, available through commercial fish importers because these fish can be tremendously difficult to identify at the species level without conducting detailed morphological analysis or EOD recordings. We compensated for restricted sample sizes wherever possible by using staggered baselines and other such strategies as described in Materials and Methods and Results. Future works will strive to conduct these experiments in the field to improve the number of animals and reduce the effects of laboratory settings.

Sample size notwithstanding, this and other recent studies (Ban et al., 2015) highlight that our understanding of electrocyte physiology is still very incomplete, especially with respect to the localization of different ion channel populations on the electrocyte membrane. Researchers have recognized the importance of the heterogeneous distribution of ion channels in electrocytes since early studies in the electric eel (Ellisman and Levinson, 1982; Fritz et al., 1983), yet little is known about the regulatory mechanisms contributing to their localization. In many cases, the density and distribution of specific ion channels is critical for the function of nerve cells, such as in the nodes of Ranvier (Schulz et al., 2008) or the axon initial segment (Kole et al., 2008), and abnormalities in channel localization can lead to neural pathologies (England et al., 1996). Further study of electrocyte ion channels in weakly electric fishes could improve our understanding of the processes governing ion channel localization as well as their evolutionary underpinnings.

In addition, these findings show the need for a better understanding of the ecology of these species and the adaptive significance of EOD plasticity. In view of *B. bennetti*'s broad geographical distribution and abundance (Crampton et al., 2016b), it seems the energetic and predation handicaps considered here have not hindered its ecological success and point to other unknown aspects of its ecology, such as mate choice preference and levels of intraspecific competition (Gavassa et al., 2012a, 2012b). Natural history studies of these animals are sorely missing and, sadly, we could be running out of time. Because of the metabolic demands of their unique sensory systems, weakly electric fishes may be disproportionately susceptible to environmental disturbances such as those induced by climate change and the rapid changes in land use currently occurring in the Amazon (Markham et al., 2016; Montag et al., 2019).
